# *N*,*N*-Dimethyl-4-[(*E*)-2-(3,6,7-tribromo-9-butyl-9*H*-carbazol-2-yl)ethen­yl]aniline

**DOI:** 10.1107/S1600536812011336

**Published:** 2012-03-21

**Authors:** Sushil Kumar, K. R. Justin Thomas, Seik Weng Ng, Edward R. T. Tiekink

**Affiliations:** a Organic Materials Laboratory, Department of Chemistry, Indian Institute of Technology Roorkee, Roorkee 247 667, India; b Department of Chemistry, University of Malaya, 50603 Kuala Lumpur, Malaysia; c Chemistry Department, Faculty of Science, King Abdulaziz University, PO Box 80203 Jeddah, Saudi Arabia

## Abstract

In the title mol­ecule, C_26_H_25_Br_3_N_2_, a dihedral angle of 6.15 (10)° is present between the carbazole and benzene ring systems with an *E* conformation about the C=C bond [1.335 (4) Å]. The butyl group is almost perpendicular to the carbazole plane [C—N—C—C torsion angle = −98.7 (3)°]. In the crystal, supra­molecular double chains along [-7,18,-16] are formed *via* C—H⋯Br and π–π inter­actions [centroid(carbazole five-membered ring)⋯centroid(carbazole six-membered ring) distance = 3.6333 (13) Å].

## Related literature

For the use of carbazole derivatives in organic light-emitting diodes and photovoltaic devices, see: Thomas *et al.* (2001 ▶, 2004 ▶); Wu *et al.* (2005 ▶); Lee *et al.* (2012 ▶); Ooyama *et al.* (2011 ▶). For related structures, see: Pawluć *et al.* (2011 ▶); Zhang & Zhang (2011 ▶); Ramathilagam *et al.* (2011 ▶).
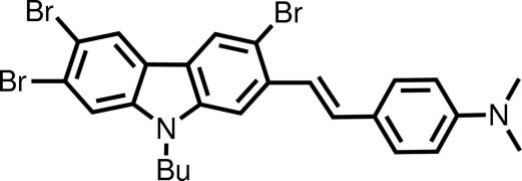


## Experimental

### 

#### Crystal data

C_26_H_25_Br_3_N_2_
*M* *_r_* = 605.21
Triclinic, 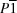

*a* = 9.7304 (3) Å
*b* = 11.3834 (4) Å
*c* = 11.8197 (4) Å
α = 114.308 (3)°
β = 101.957 (3)°
γ = 90.127 (3)°
*V* = 1161.62 (7) Å^3^
*Z* = 2
Cu *K*α radiation
μ = 6.56 mm^−1^
*T* = 100 K
0.30 × 0.30 × 0.05 mm

#### Data collection

Agilent SuperNova Dual diffractometer with an Atlas detector
Absorption correction: multi-scan (*CrysAlis PRO*; Agilent, 2010 ▶) *T*_min_ = 0.244, *T*_max_ = 0.735
10766 measured reflections
4827 independent reflections
4680 reflections with *I* > 2σ(*I*)
*R*_int_ = 0.021

#### Refinement

*R*[*F* ^2^ > 2σ(*F* ^2^)] = 0.032
*wR*(*F* ^2^) = 0.092
*S* = 1.07
4827 reflections
282 parameters
H-atom parameters constrained
Δρ_max_ = 1.18 e Å^−3^
Δρ_min_ = −0.81 e Å^−3^

### 

Data collection: *CrysAlis PRO* (Agilent, 2010 ▶); cell refinement: *CrysAlis PRO*; data reduction: *CrysAlis PRO*; program(s) used to solve structure: *SHELXS97* (Sheldrick, 2008 ▶); program(s) used to refine structure: *SHELXL97* (Sheldrick, 2008 ▶); molecular graphics: *ORTEP-3* (Farrugia, 1997 ▶) and *DIAMOND* (Brandenburg, 2006 ▶); software used to prepare material for publication: *publCIF* (Westrip, 2010 ▶).

## Supplementary Material

Crystal structure: contains datablock(s) global, I. DOI: 10.1107/S1600536812011336/gg2078sup1.cif

Structure factors: contains datablock(s) I. DOI: 10.1107/S1600536812011336/gg2078Isup2.hkl

Supplementary material file. DOI: 10.1107/S1600536812011336/gg2078Isup3.cml

Additional supplementary materials:  crystallographic information; 3D view; checkCIF report

## Figures and Tables

**Table 1:** Hydrogen-bond geometry (Å, °)

*D*—H⋯*A*	*D*—H	H⋯*A*	*D*⋯*A*	*D*—H⋯*A*
C26—H26*C*⋯Br1^i^	0.98	2.91	3.844 (3)	161

Symmetry code: (i) 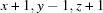.

**Fig. 1.**
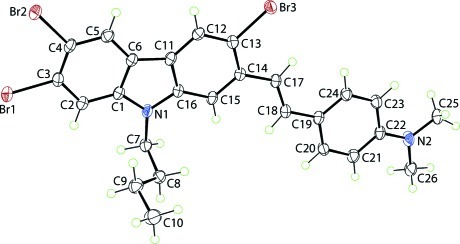
The molecular structure of (I) showing the atom-labelling scheme and displacement ellipsoids at the 50% probability level.

**Fig. 2.**
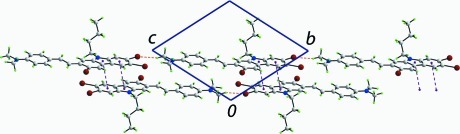
A view of the linear supramolecular chain in (I). The C—H···Br and π–π interactions are shown as orange and purple dashed lines, respectively.

**Fig. 3.**
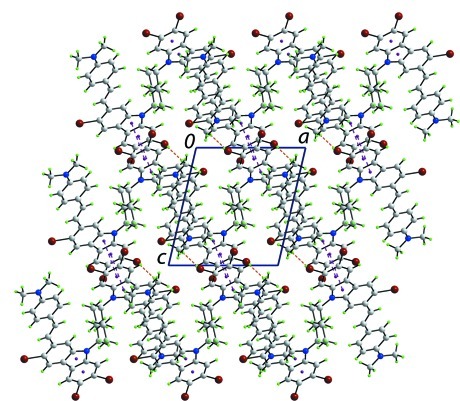
A view in projection down the b axis of the stacking of layers, formed by non-interacting supramolecular chains, in (I). The C—H···Br and π–π interactions are shown as orange and purple dashed lines, respectively.

## supplementary crystallographic information

### Comment

Polysubstituted carbazole derivatives have been widely explored as functional materials for applications in organic light-emitting diodes (Thomas *et al.*, 2001; Thomas *et al.*, 2004; Wu *et al.*, 2005) and photovoltaic devices (Lee *et al.*, 2012; Ooyama *et al.*, 2011) due to their charge transporting and amorphous properties. Though the 3,6,9-trisubstituted (Thomas *et al.*, 2001; Thomas *et al.*, 2004) and 2,7,9-trisubstituted carbazole (Wu *et al.*, 2005; Lee *et al.*, 2012) compounds have been well documented in the literature, 2,3,6,7,9-pentasubstituted carbazole derivatives are relatively rare. Herein, the synthesis and crystal structure determination of the title compound, (I), are described. Several related structures are known (Pawluć *et al.*, 2011; Zhang & Zhang, 2011; Ramathilagam *et al.*, 2011).

In (I), the carbazole fused-ring system is planar with the r.m.s. deviation of the 13 fitted non-hydrogen atoms = 0.006 Å; the Br1, Br2 and Br3 atoms lie 0.058 (1), 0.062 (1) and 0.043 (1) Å out of this plane, respectively. The least-squares plane through the carbazole residue forms a dihedral angle of 6.15 (10)° with the benzene ring, indicating a small twist between the terminal ring systems. This twist is manifested in the value of the C15—C14—C17—C18 torsion angle of -11.2 (4)°. The butyl group is almost perpendicular to the carbazole plane with the C1—N1—C7—C8 torsion angle being -98.7 (3)°. Finally, the conformation about the C17═C18 bond [1.335 (4) Å] is *E*.

In the crystal packing, molecules are linked into linear supramolecular chains *via* C—H···Br interactions, Fig. 2 and Table 1. These are connected into double chains along [7 18 16] *via*π–π interactions occurring between five- and six-membered rings of the carbazole residue [centroid(N1,C1,C6,C11,C16)···centroid(C1–C6)^i^ = 3.6333 (13) Å, angle between rings = 0.50 (12)° for symmetry operation *i*: 1 - *x*, 1 - *y*, -*z*]. Chains assemble into layers, with no specific interactions between them. In turn, the layers stack along (2 0 2), again without specific interactions between them, Fig. 2.

### Experimental

A mixture of 2,3,6,7-tetrabromo-9-butyl-9*H*-carbazole (0.25 g, 0.47 mmol), styrene (0.29 g, 1.96 mmol), tetrabutylammonium bromide (0.32 g, 0.98 mmol), sodium acetate (1.6 g, 19.6 mmol), Pd(OAc)_2_ (4 mg) and dimethylformamide (5 ml) was heated at 383 K for 48 h. Subsequently, it was cooled, then poured into water and extracted using ethyl acetate. On removal of solvent, a residue was obtained which on purification by column chromatography on silica gel gave an orange crystalline solid. Yield: 0.12 g, 42%. *M*.pt: 414 K. Crystals were grown from a solution of the title compound dissolved in dichloromethane/hexanes mixture (1:9 *v*/*v*).

^1^H NMR (500 MHz, CDCl_3_) δ: 8.20 (s, 1 H), 8.17 (s, 1 H), 7.63 (s, 1H), 7.58 (s, 1 H), 7.51 (d, J = 9.0 Hz, 2 H), 7.43 (d, J = 16 Hz, 1 H), 7.05 (d, J = 16 Hz, 1 H), 6.74 (d, J = 9 Hz, 2 H), 4.24 (t, J = 7.5 Hz, 2 H), 3.02 (s, 6 H), 1.86–1.83 (m, 2 H), 1.42–1.37 (m, 2 H), 0.97 (t, J = 7.5 Hz, 3 H); ^13^C NMR (125 MHz, CDCl_3_) δ: 150.4, 140.7, 140.5, 136.2, 131.4, 128.1, 127.8, 127.4, 125.4, 124.6, 124.4, 124.0, 122.7, 121.6, 121.4, 115.0, 114.0, 113.5, 112.5, 112.4, 105.8, 100.0, 43.2, 40.5, 30.9, 20.6, 13.9.

### Refinement

Carbon-bound H-atoms were placed in calculated positions [C—H = 0.95 to 0.99 Å, *U*_iso_(H) = 1.2 to 1.5*U*_eq_(C)] and were included in the refinement in the riding model approximation. The maximum and minimum residual electron density peaks of 1.18 and 0.81 e Å^-3^, respectively, were located 0.86 Å and 0.44 Å from the H2 and Br1 atoms, respectively.

### Crystal data

**Table:**

C_26_H_25_Br_3_N_2_	*Z* = 2
*M**_r_* = 605.21	*F*(000) = 600
Triclinic, *P*1	*D*_x_ = 1.730 Mg m^−^^3^
Hall symbol: -P 1	Cu *K*α radiation, λ = 1.54184 Å
*a* = 9.7304 (3) Å	Cell parameters from 7879 reflections
*b* = 11.3834 (4) Å	θ = 4.2–76.4°
*c* = 11.8197 (4) Å	µ = 6.56 mm^−^^1^
α = 114.308 (3)°	*T* = 100 K
β = 101.957 (3)°	Plate, orange
γ = 90.127 (3)°	0.30 × 0.30 × 0.05 mm
*V* = 1161.62 (7) Å^3^	

### Data collection

**Table:**

Agilent SuperNova Dual diffractometer with an Atlas detector	4827 independent reflections
Radiation source: SuperNova (Cu) X-ray Source	4680 reflections with *I* > 2σ(*I*)
Mirror monochromator	*R*_int_ = 0.021
Detector resolution: 10.4041 pixels mm^-1^	θ_max_ = 76.6°, θ_min_ = 4.2°
ω scan	*h* = −12→12
Absorption correction: multi-scan (*CrysAlis PRO*; Agilent, 2010)	*k* = −14→14
*T*_min_ = 0.244, *T*_max_ = 0.735	*l* = −14→10
10766 measured reflections	

### Refinement

**Table:**

Refinement on *F*^2^	Primary atom site location: structure-invariant direct methods
Least-squares matrix: full	Secondary atom site location: difference Fourier map
*R*[*F*^2^ > 2σ(*F*^2^)] = 0.032	Hydrogen site location: inferred from neighbouring sites
*wR*(*F*^2^) = 0.092	H-atom parameters constrained
*S* = 1.07	*w* = 1/[σ^2^(*F*_o_^2^) + (0.0613*P*)^2^ + 0.89*P*] where *P* = (*F*_o_^2^ + 2*F*_c_^2^)/3
4827 reflections	(Δ/σ)_max_ = 0.001
282 parameters	Δρ_max_ = 1.18 e Å^−^^3^
0 restraints	Δρ_min_ = −0.81 e Å^−^^3^

### Special details

**Table:**

Geometry. All e.s.d.'s (except the e.s.d. in the dihedral angle between two l.s. planes) are estimated using the full covariance matrix. The cell e.s.d.'s are taken into account individually in the estimation of e.s.d.'s in distances, angles and torsion angles; correlations between e.s.d.'s in cell parameters are only used when they are defined by crystal symmetry. An approximate (isotropic) treatment of cell e.s.d.'s is used for estimating e.s.d.'s involving l.s. planes.
Refinement. Refinement of *F*^2^ against ALL reflections. The weighted *R*-factor *wR* and goodness of fit *S* are based on *F*^2^, conventional *R*-factors *R* are based on *F*, with *F* set to zero for negative *F*^2^. The threshold expression of *F*^2^ > σ(*F*^2^) is used only for calculating *R*-factors(gt) *etc*. and is not relevant to the choice of reflections for refinement. *R*-factors based on *F*^2^ are statistically about twice as large as those based on *F*, and *R*- factors based on ALL data will be even larger.

### Fractional atomic coordinates and isotropic or equivalent isotropic displacement parameters (Å^2^)

**Table:**

	*x*	*y*	*z*	*U*_iso_*/*U*_eq_	
Br1	0.29708 (3)	0.83996 (3)	0.02986 (2)	0.02733 (9)	
Br2	0.56802 (3)	0.78783 (3)	−0.11524 (2)	0.02739 (9)	
Br3	1.11706 (3)	0.35337 (3)	0.20455 (3)	0.02790 (9)	
N1	0.5643 (2)	0.5740 (2)	0.27349 (19)	0.0222 (4)	
N2	1.1308 (3)	0.0298 (3)	0.7703 (2)	0.0333 (5)	
C1	0.5457 (2)	0.6291 (2)	0.1869 (2)	0.0209 (4)	
C2	0.4366 (3)	0.6979 (2)	0.1578 (2)	0.0228 (5)	
H2	0.3598	0.7132	0.1986	0.027*	
C3	0.4443 (3)	0.7433 (2)	0.0664 (2)	0.0226 (5)	
C4	0.5569 (3)	0.7208 (2)	0.0055 (2)	0.0227 (5)	
C5	0.6653 (3)	0.6523 (2)	0.0351 (2)	0.0232 (5)	
H5	0.7418	0.6373	−0.0061	0.028*	
C6	0.6601 (2)	0.6056 (2)	0.1262 (2)	0.0205 (4)	
C7	0.4735 (2)	0.5794 (2)	0.3587 (2)	0.0236 (5)	
H7A	0.4651	0.4940	0.3615	0.028*	
H7B	0.3780	0.5967	0.3240	0.028*	
C8	0.5283 (3)	0.6836 (2)	0.4944 (2)	0.0263 (5)	
H8A	0.4684	0.6752	0.5494	0.032*	
H8B	0.6253	0.6681	0.5274	0.032*	
C9	0.5301 (3)	0.8209 (3)	0.5043 (2)	0.0295 (5)	
H9A	0.4321	0.8398	0.4793	0.035*	
H9B	0.5833	0.8280	0.4441	0.035*	
C10	0.5972 (3)	0.9205 (3)	0.6384 (3)	0.0357 (6)	
H10A	0.5967	1.0074	0.6403	0.054*	
H10B	0.6947	0.9029	0.6632	0.054*	
H10C	0.5434	0.9155	0.6980	0.054*	
C11	0.7525 (3)	0.5336 (2)	0.1799 (2)	0.0213 (4)	
C12	0.8799 (3)	0.4835 (2)	0.1613 (2)	0.0223 (5)	
H12	0.9246	0.4940	0.1014	0.027*	
C13	0.9406 (2)	0.4177 (2)	0.2322 (2)	0.0217 (4)	
C14	0.8789 (2)	0.3989 (2)	0.3234 (2)	0.0207 (4)	
C15	0.7510 (3)	0.4504 (2)	0.3406 (2)	0.0216 (4)	
H15	0.7059	0.4402	0.4004	0.026*	
C16	0.6895 (2)	0.5168 (2)	0.2704 (2)	0.0207 (4)	
C17	0.9466 (2)	0.3259 (2)	0.3949 (2)	0.0211 (4)	
H17	1.0239	0.2799	0.3683	0.025*	
C18	0.9070 (3)	0.3201 (2)	0.4942 (2)	0.0238 (5)	
H18	0.8316	0.3687	0.5218	0.029*	
C19	0.9685 (3)	0.2460 (2)	0.5642 (2)	0.0222 (5)	
C20	0.9072 (3)	0.2399 (3)	0.6593 (2)	0.0258 (5)	
H20	0.8267	0.2851	0.6769	0.031*	
C21	0.9600 (3)	0.1702 (3)	0.7288 (3)	0.0274 (5)	
H21	0.9152	0.1683	0.7923	0.033*	
C22	1.0787 (3)	0.1029 (2)	0.7058 (2)	0.0245 (5)	
C23	1.1426 (3)	0.1108 (2)	0.6119 (2)	0.0253 (5)	
H23	1.2248	0.0679	0.5958	0.030*	
C24	1.0881 (3)	0.1797 (2)	0.5433 (2)	0.0243 (5)	
H24	1.1331	0.1822	0.4801	0.029*	
C25	1.2647 (3)	−0.0235 (3)	0.7574 (3)	0.0300 (5)	
H25A	1.3375	0.0461	0.7772	0.045*	
H25B	1.2562	−0.0885	0.6698	0.045*	
H25C	1.2909	−0.0641	0.8166	0.045*	
C26	1.0606 (3)	0.0186 (3)	0.8627 (3)	0.0328 (6)	
H26A	0.9602	−0.0093	0.8233	0.049*	
H26B	1.0708	0.1029	0.9360	0.049*	
H26C	1.1033	−0.0453	0.8912	0.049*	

### Atomic displacement parameters (Å^2^)

**Table:**

	*U*^11^	*U*^22^	*U*^33^	*U*^12^	*U*^13^	*U*^23^
Br1	0.02941 (16)	0.02848 (15)	0.02854 (15)	0.00817 (11)	0.00398 (11)	0.01757 (11)
Br2	0.03297 (16)	0.03048 (15)	0.02791 (15)	0.00781 (11)	0.00784 (11)	0.02082 (12)
Br3	0.02538 (15)	0.03522 (16)	0.03272 (16)	0.01107 (11)	0.01128 (11)	0.02151 (12)
N1	0.0211 (9)	0.0276 (10)	0.0239 (9)	0.0051 (8)	0.0045 (8)	0.0172 (8)
N2	0.0314 (12)	0.0441 (13)	0.0403 (13)	0.0144 (10)	0.0105 (10)	0.0322 (11)
C1	0.0224 (11)	0.0212 (10)	0.0206 (10)	0.0004 (9)	0.0019 (8)	0.0117 (9)
C2	0.0244 (11)	0.0240 (11)	0.0226 (11)	0.0030 (9)	0.0040 (9)	0.0130 (9)
C3	0.0235 (11)	0.0209 (10)	0.0229 (11)	0.0036 (9)	0.0010 (9)	0.0109 (9)
C4	0.0295 (12)	0.0206 (10)	0.0194 (10)	0.0024 (9)	0.0017 (9)	0.0117 (9)
C5	0.0274 (12)	0.0224 (11)	0.0224 (11)	0.0022 (9)	0.0060 (9)	0.0119 (9)
C6	0.0229 (11)	0.0194 (10)	0.0196 (10)	0.0016 (8)	0.0024 (8)	0.0097 (9)
C7	0.0200 (11)	0.0291 (12)	0.0287 (12)	0.0031 (9)	0.0068 (9)	0.0184 (10)
C8	0.0274 (12)	0.0322 (13)	0.0270 (12)	0.0070 (10)	0.0095 (9)	0.0184 (10)
C9	0.0344 (13)	0.0315 (13)	0.0281 (12)	0.0033 (10)	0.0084 (10)	0.0172 (11)
C10	0.0428 (16)	0.0347 (14)	0.0303 (13)	0.0040 (12)	0.0104 (12)	0.0133 (11)
C11	0.0242 (11)	0.0199 (10)	0.0212 (11)	0.0006 (9)	0.0022 (9)	0.0116 (9)
C12	0.0254 (12)	0.0231 (11)	0.0211 (11)	0.0018 (9)	0.0051 (9)	0.0120 (9)
C13	0.0193 (11)	0.0225 (10)	0.0238 (11)	0.0021 (8)	0.0035 (9)	0.0109 (9)
C14	0.0198 (11)	0.0218 (11)	0.0216 (11)	0.0014 (8)	0.0024 (8)	0.0112 (9)
C15	0.0238 (11)	0.0227 (11)	0.0230 (11)	0.0029 (9)	0.0037 (9)	0.0150 (9)
C16	0.0203 (11)	0.0217 (10)	0.0215 (11)	0.0023 (8)	0.0020 (8)	0.0118 (9)
C17	0.0206 (10)	0.0193 (10)	0.0239 (11)	0.0013 (8)	0.0018 (8)	0.0112 (9)
C18	0.0230 (11)	0.0240 (11)	0.0262 (11)	0.0056 (9)	0.0028 (9)	0.0137 (9)
C19	0.0214 (11)	0.0235 (11)	0.0240 (11)	0.0017 (9)	0.0023 (9)	0.0136 (9)
C20	0.0220 (11)	0.0318 (12)	0.0301 (12)	0.0086 (9)	0.0080 (9)	0.0183 (10)
C21	0.0257 (12)	0.0344 (13)	0.0292 (12)	0.0037 (10)	0.0069 (10)	0.0200 (11)
C22	0.0234 (11)	0.0261 (11)	0.0285 (12)	0.0036 (9)	0.0021 (9)	0.0177 (10)
C23	0.0228 (11)	0.0272 (12)	0.0303 (12)	0.0061 (9)	0.0061 (9)	0.0164 (10)
C24	0.0238 (11)	0.0262 (12)	0.0274 (12)	0.0045 (9)	0.0055 (9)	0.0160 (10)
C25	0.0283 (13)	0.0314 (13)	0.0345 (13)	0.0084 (10)	0.0028 (10)	0.0200 (11)
C26	0.0347 (14)	0.0421 (15)	0.0350 (14)	0.0110 (12)	0.0093 (11)	0.0286 (12)

### Geometric parameters (Å, º)

**Table:**

Br1—C3	1.895 (2)	C11—C12	1.386 (3)
Br2—C4	1.895 (2)	C11—C16	1.413 (3)
Br3—C13	1.906 (2)	C12—C13	1.388 (3)
N1—C16	1.382 (3)	C12—H12	0.9500
N1—C1	1.388 (3)	C13—C14	1.423 (3)
N1—C7	1.455 (3)	C14—C15	1.394 (3)
N2—C22	1.378 (3)	C14—C17	1.479 (3)
N2—C26	1.447 (3)	C15—C16	1.389 (3)
N2—C25	1.448 (3)	C15—H15	0.9500
C1—C2	1.390 (3)	C17—C18	1.335 (4)
C1—C6	1.415 (3)	C17—H17	0.9500
C2—C3	1.390 (3)	C18—C19	1.458 (3)
C2—H2	0.9500	C18—H18	0.9500
C3—C4	1.401 (4)	C19—C24	1.398 (3)
C4—C5	1.385 (3)	C19—C20	1.401 (3)
C5—C6	1.393 (3)	C20—C21	1.390 (4)
C5—H5	0.9500	C20—H20	0.9500
C6—C11	1.442 (3)	C21—C22	1.399 (4)
C7—C8	1.530 (4)	C21—H21	0.9500
C7—H7A	0.9900	C22—C23	1.412 (3)
C7—H7B	0.9900	C23—C24	1.379 (3)
C8—C9	1.518 (4)	C23—H23	0.9500
C8—H8A	0.9900	C24—H24	0.9500
C8—H8B	0.9900	C25—H25A	0.9800
C9—C10	1.520 (4)	C25—H25B	0.9800
C9—H9A	0.9900	C25—H25C	0.9800
C9—H9B	0.9900	C26—H26A	0.9800
C10—H10A	0.9800	C26—H26B	0.9800
C10—H10B	0.9800	C26—H26C	0.9800
C10—H10C	0.9800		
			
C16—N1—C1	108.3 (2)	C11—C12—C13	118.5 (2)
C16—N1—C7	124.7 (2)	C11—C12—H12	120.7
C1—N1—C7	126.9 (2)	C13—C12—H12	120.7
C22—N2—C26	120.0 (2)	C12—C13—C14	123.4 (2)
C22—N2—C25	120.1 (2)	C12—C13—Br3	116.64 (18)
C26—N2—C25	119.5 (2)	C14—C13—Br3	119.95 (18)
C2—C1—N1	129.2 (2)	C15—C14—C13	117.0 (2)
C2—C1—C6	121.6 (2)	C15—C14—C17	121.7 (2)
N1—C1—C6	109.1 (2)	C13—C14—C17	121.3 (2)
C1—C2—C3	117.4 (2)	C14—C15—C16	120.1 (2)
C1—C2—H2	121.3	C14—C15—H15	119.9
C3—C2—H2	121.3	C16—C15—H15	119.9
C2—C3—C4	121.7 (2)	N1—C16—C15	128.7 (2)
C2—C3—Br1	117.04 (19)	N1—C16—C11	109.5 (2)
C4—C3—Br1	121.27 (18)	C15—C16—C11	121.8 (2)
C5—C4—C3	120.7 (2)	C18—C17—C14	124.7 (2)
C5—C4—Br2	118.33 (19)	C18—C17—H17	117.7
C3—C4—Br2	120.98 (18)	C14—C17—H17	117.7
C4—C5—C6	118.8 (2)	C17—C18—C19	126.1 (2)
C4—C5—H5	120.6	C17—C18—H18	117.0
C6—C5—H5	120.6	C19—C18—H18	117.0
C5—C6—C1	119.8 (2)	C24—C19—C20	116.7 (2)
C5—C6—C11	133.5 (2)	C24—C19—C18	123.8 (2)
C1—C6—C11	106.6 (2)	C20—C19—C18	119.5 (2)
N1—C7—C8	112.9 (2)	C21—C20—C19	122.3 (2)
N1—C7—H7A	109.0	C21—C20—H20	118.8
C8—C7—H7A	109.0	C19—C20—H20	118.8
N1—C7—H7B	109.0	C20—C21—C22	120.4 (2)
C8—C7—H7B	109.0	C20—C21—H21	119.8
H7A—C7—H7B	107.8	C22—C21—H21	119.8
C9—C8—C7	114.0 (2)	N2—C22—C21	121.7 (2)
C9—C8—H8A	108.8	N2—C22—C23	120.8 (2)
C7—C8—H8A	108.8	C21—C22—C23	117.5 (2)
C9—C8—H8B	108.8	C24—C23—C22	121.3 (2)
C7—C8—H8B	108.8	C24—C23—H23	119.4
H8A—C8—H8B	107.7	C22—C23—H23	119.4
C8—C9—C10	112.2 (2)	C23—C24—C19	121.7 (2)
C8—C9—H9A	109.2	C23—C24—H24	119.1
C10—C9—H9A	109.2	C19—C24—H24	119.1
C8—C9—H9B	109.2	N2—C25—H25A	109.5
C10—C9—H9B	109.2	N2—C25—H25B	109.5
H9A—C9—H9B	107.9	H25A—C25—H25B	109.5
C9—C10—H10A	109.5	N2—C25—H25C	109.5
C9—C10—H10B	109.5	H25A—C25—H25C	109.5
H10A—C10—H10B	109.5	H25B—C25—H25C	109.5
C9—C10—H10C	109.5	N2—C26—H26A	109.5
H10A—C10—H10C	109.5	N2—C26—H26B	109.5
H10B—C10—H10C	109.5	H26A—C26—H26B	109.5
C12—C11—C16	119.2 (2)	N2—C26—H26C	109.5
C12—C11—C6	134.4 (2)	H26A—C26—H26C	109.5
C16—C11—C6	106.4 (2)	H26B—C26—H26C	109.5
			
C16—N1—C1—C2	−179.3 (2)	C12—C13—C14—C17	178.9 (2)
C7—N1—C1—C2	−2.2 (4)	Br3—C13—C14—C17	−2.1 (3)
C16—N1—C1—C6	0.7 (3)	C13—C14—C15—C16	−0.1 (3)
C7—N1—C1—C6	177.8 (2)	C17—C14—C15—C16	−179.1 (2)
N1—C1—C2—C3	179.8 (2)	C1—N1—C16—C15	180.0 (2)
C6—C1—C2—C3	−0.2 (4)	C7—N1—C16—C15	2.7 (4)
C1—C2—C3—C4	0.2 (4)	C1—N1—C16—C11	−0.6 (3)
C1—C2—C3—Br1	−178.73 (17)	C7—N1—C16—C11	−177.9 (2)
C2—C3—C4—C5	−0.2 (4)	C14—C15—C16—N1	179.8 (2)
Br1—C3—C4—C5	178.67 (18)	C14—C15—C16—C11	0.4 (4)
C2—C3—C4—Br2	−178.20 (19)	C12—C11—C16—N1	180.0 (2)
Br1—C3—C4—Br2	0.6 (3)	C6—C11—C16—N1	0.4 (3)
C3—C4—C5—C6	0.2 (4)	C12—C11—C16—C15	−0.6 (3)
Br2—C4—C5—C6	178.27 (18)	C6—C11—C16—C15	179.8 (2)
C4—C5—C6—C1	−0.2 (4)	C15—C14—C17—C18	−11.2 (4)
C4—C5—C6—C11	−179.3 (2)	C13—C14—C17—C18	169.8 (2)
C2—C1—C6—C5	0.2 (4)	C14—C17—C18—C19	178.0 (2)
N1—C1—C6—C5	−179.8 (2)	C17—C18—C19—C24	6.3 (4)
C2—C1—C6—C11	179.5 (2)	C17—C18—C19—C20	−174.2 (2)
N1—C1—C6—C11	−0.4 (3)	C24—C19—C20—C21	−1.0 (4)
C16—N1—C7—C8	78.0 (3)	C18—C19—C20—C21	179.5 (2)
C1—N1—C7—C8	−98.7 (3)	C19—C20—C21—C22	0.2 (4)
N1—C7—C8—C9	65.2 (3)	C26—N2—C22—C21	1.4 (4)
C7—C8—C9—C10	−175.0 (2)	C25—N2—C22—C21	−171.3 (2)
C5—C6—C11—C12	−0.2 (5)	C26—N2—C22—C23	−177.6 (3)
C1—C6—C11—C12	−179.5 (3)	C25—N2—C22—C23	9.7 (4)
C5—C6—C11—C16	179.3 (3)	C20—C21—C22—N2	−178.0 (3)
C1—C6—C11—C16	0.0 (3)	C20—C21—C22—C23	1.1 (4)
C16—C11—C12—C13	0.4 (3)	N2—C22—C23—C24	177.5 (3)
C6—C11—C12—C13	179.8 (2)	C21—C22—C23—C24	−1.6 (4)
C11—C12—C13—C14	0.0 (4)	C22—C23—C24—C19	0.8 (4)
C11—C12—C13—Br3	−179.05 (17)	C20—C19—C24—C23	0.6 (4)
C12—C13—C14—C15	−0.2 (4)	C18—C19—C24—C23	180.0 (2)
Br3—C13—C14—C15	178.85 (17)		

### Hydrogen-bond geometry (Å, º)

**Table:**

*D*—H···*A*	*D*—H	H···*A*	*D*···*A*	*D*—H···*A*
C26—H26*C*···Br1^i^	0.98	2.91	3.844 (3)	161

Symmetry code: (i) *x*+1, *y*−1, *z*+1.
